# Trastuzumab in the adjuvant setting: concurrent or sequential? It takes two to tango!

**DOI:** 10.3747/co.2008.204

**Published:** 2008-01

**Authors:** Sunil Verma

**Affiliations:** Toronto–Sunnybrook Regional Cancer Centre, Toronto, Ontario

## CASE STUDY

Two months ago, on routine physical examination by family physician, a 58-year-old woman was diagnosed with a breast lump. She received appropriate imaging, and a core biopsy confirmed invasive ductal carcinoma that was hormone receptor–negative and 3+ by immunohistochemistry for human epidermal growth factor-2 (her2). Five weeks ago, the woman underwent lumpectomy, and the final pathology report recorded a 2.4-cm tumour, grade III with lymphovascular invasion. One sentinel lymph node was positive, and the remaining 11 nodes on the subsequent axillary nodal dissection were negative, for a total count of 1 of 12 lymph nodes involved. This woman is otherwise healthy and is not taking any medications. Her staging workup was negative. A baseline multiple gated acquisition scan showed an ejection fraction (ef) of 58%.

What trastuzumab-based regimen would you suggest for this patient? A concurrent or sequential approach?

## DISCUSSION

### Which Approach Is the Most Cardiac-safe?

More than 13,000 patients have been enrolled in trastuzumab adjuvant trials so far, but the longest cardiac follow-up to date is only 5 years. A look at the adjuvant trastuzumab studies shows rates of grades 3 and 4 cardiac toxicity that range from 0.4% [with the Breast Cancer International Research Group 006 docetaxel–carboplatin–trastuzumab (tch) regimen] to up to 4% (with a concurrent regimen containing an anthracycline and a taxane, combined analysis) 1. The hera (Herceptin Adjuvant) trial reports a 0.6% rate of symptomatic congestive heart failure with sequential trastuzumab; however, their patient population was notably quite selective. The patients had to have finished chemotherapy and then had to have an ef above 55% to be eligible for randomization. Also, patients experienced a median delay of about 8 months from surgery to first dose of trastuzumab and also a significant delay from completion of the anthracycline to the start of trastuzumab.

Because these individual trials were quite different in their eligibility criteria, cardiac follow-up recommendations, and endpoint definitions, comparing their cardiac toxicity results is quite difficult. The only trial that is truly comparing concurrent and sequential approaches to trastuzumab is the North Central Cancer Treatment Group (ncctg) N9831 trial. The updated 3-year cardiac follow-up of that trial now shows a cardiac event rate of 2.8% in the sequential trastuzumab arm as compared with 3.3% in the concurrent arm 2, a difference that is not as marked as initially thought—likely less than 1%.

It does appear that a non-anthracycline trastuzumab protocol—that is, tch—is the most cardiac-safe. Furthermore, there is no significant difference in cardiac toxicity between the concurrent and sequential approaches.

### Which Approach Is the Most Effective?

The key trial that will answer the question of effectiveness is ncctg N9831, which randomized patients to no trastuzumab, concurrent trastuzumab, or sequential trastuzumab with a chemotherapy backbone of cyclophosphamide–doxorubicin (ac) followed by weekly paclitaxel. The results of the unplanned interim analysis presented at the 2005 American Society of Clinical Oncology meeting demonstrated that the concurrent approach was superior to the sequential approach, and furthermore, that the sequential approach was no better than control. However, given that the interim analysis was unplanned, most clinicians will not make treatment decisions based on its results.

Another trial presented at the 2007 San Antonio Breast Cancer Symposium also indicated that the sequential approach may not be as effective. The randomized pacs-04 study was initially designed to compare 6 cycles of fluorouracil–epirubicin–cyclophosphamide (fec100) to 6 cycles of epirubicin given concurrently with docetaxel 3. Then, in a second randomization, her2-positive patients were randomized to receive or not receive trastuzumab after completion of chemotherapy. The authors presented their results with a median follow-up of 4 years. No significant improvement was observed in disease-free survival [hazard ratio (hr): 0.86; *p* = 0.41] or overall survival (hr: 1.27; *p* = not available). There did appear to be initial efficacy in the first 18 months, but the effect was lost over the next 2 years. Numerically, fewer metastatic events were seen on the trastuzumab arm.

And so pacs-04 is the first negative adjuvant trastuzumab trial. Why, given all the previous trials, was an additional benefit of trastuzumab not seen in its patients?

The pacs-04 patients all received adequate anthracyclines, and about 50% had an adequate dose of a taxane. The results seen are similar to those from the unplanned analysis of the sequential arm of ncctg N9831 [hazard ratio (hr): 0.87]. Also, it is concerning that, with sequential therapy, a benefit initially seen is lost with longer follow-up. The suggestion is that trastuzumab may have a cytostatic effect when given sequentially as compared with a cytotoxic effect when given concurrently.

Why was no benefit seen with the sequential approach in the pacs-04 trial, when a benefit was observed in hera (Table I) 3,4? One explanation may be that the patients in hera did not receive adequate chemotherapy, and that in the presence of third-generation regimen, the benefit of trastuzumab is seen only in a concurrent and not in a sequential regimen.

The evidence from concurrent regimens has previously showed a benefit in the range of about 50% reduction in the relative risk of disease recurrence and 40% reduction in the relative risk of mortality. And notably, these results occurred despite a backbone of third-generation chemotherapy regimens such as ac followed by paclitaxel or by docetaxel.

### What Should the Recommendation Be for the Current Patient?

Early data appear to indicate that a concurrent approach is more efficacious than a sequential approach. Furthermore, the additional cardiac toxicity appears not to be quite as marked as initially thought, and may be even further mitigated with the use of a non-anthracycline-based concurrent regimen. The key ncctg N9831 trial results are eagerly awaited, and their availability later in 2008 will probably settle this debate.

Coming back to the specific patient, I suggest that she be offered a concurrent trastuzumab regimen with appropriate cardiac follow-up.

## Figures and Tables

**TABLE I:** Sequential approach with trastuzumab: hera compared with pacs-04

	*Trials*	
	hera	pacs*-04*
Median follow-up (years)	2	4
Anthracyclines (%)	96	100
Anthracyclines and taxanes (%)	26	50
Node-positive (%)	32	100
Disease-free survival	0.64	0.86
	(*p<*0.0001)	(*p=*0.41)
Overall survival	0.66	1.27
	(*p=*0.0115)	(*p=*na)

na = not available.
